# Centrality Analysis Methods for Biological Networks and Their Application to Gene Regulatory Networks

**DOI:** 10.4137/grsb.s702

**Published:** 2008-05-15

**Authors:** Dirk Koschützki, Falk Schreiber

**Affiliations:** 1 Department of Molecular Genetics, Leibniz Institute of Plant Genetics and Crop Plant Research (IPK), Corrensstraße 3, 06466 Gatersleben, Germany; 2 Department of Computer and Electrical Engineering, Furtwangen University of Applied Sciences, Robert-Gerwig-Platz 1, 78120 Furtwangen, Germany; 3 Institute for Computer Science, Martin-Luther-University Halle-Wittenberg, Von-Seckendorff-Platz 1, 06120 Halle, Germany

**Keywords:** network analysis, centralities, gene regulatory network, escherichia coli, network motif

## Abstract

The structural analysis of biological networks includes the ranking of the vertices based on the connection structure of a network. To support this analysis we discuss centrality measures which indicate the importance of vertices, and demonstrate their applicability on a gene regulatory network. We show that common centrality measures result in different valuations of the vertices and that novel measures tailored to specific biological investigations are useful for the analysis of biological networks, in particular gene regulatory networks.

## Introduction

The interaction of biological entities such as genes, proteins and metabolites is of great interest in life science research and is increasingly important for systems biological approaches (Oltvai and Barabási (2002); Kitano (2002)). The interplay of different interactions is often represented by biological networks such as gene regulatory, protein interaction and metabolic networks. To investigate these complex and large networks different network analysis methods have been developed or employed from other fields of sciences (Junker and Schreiber (2008)). Centrality analysis, the ranking of network elements used to identify interesting elements of a network is one of these methods (Koschützki et al. (2005)). It is particularly useful to identify key players in biological processes. For example, it has been shown that highly connected vertices in protein interaction networks are often functionally important and the deletion of such vertices is related to lethality (Jeong et al. (2001)). Wuchty and Stadler applied three different types of centralities to metabolic, protein interaction and domain sequence networks (Wuchty and Stadler (2003)). Fell and Wagner discuss the possibility that metabolites with highest degree (i.e. highest number of connections) may belong to the oldest part of the metabolism (Fell and Wagner (2000)). However, it has also been shown that the degree of a vertex alone, as a specific centrality measure, is not sufficient to distinguish lethal proteins clearly from viable ones (Wuchty (2002)), that in protein networks there is no relation between network connectivity and robustness against aminoacid substitutions (Hahn et al. (2004)), and that for biological network analysis several centrality measures have to be considered (Wuchty and Stadler (2003); Koschützki and Schreiber (2004)).

To assist scientists in the exploration of biological networks, we discuss and compare different centrality measures. Some of them are already known in biological sciences, others are transferred from different fields of sciences such as social network analysis. We also show that it is useful to consider biological knowledge in network analysis and discuss motif-based centralities which have been specifically developed for gene regulatory networks.

## Graphs and Centralities

A network is an informal description for a set of elements with connections between them. In a formal way a network is modelled as a mathematical object called graph. A directed graph *G* = (*V*, *E*) consists of a finite set *V* of vertices and a finite set *E* ⊆ *V* × *V* of directed edges. An edge *e* = (*u*, *v*) connects two vertices *u* and *v* and is directed from *u* to *v*. The vertices *u* and *v* are said to be incident with the edge *e* and adjacent to each other. The set of all vertices which are adjacent to a vertex *u* is called the neighbourhood *N*(*u*) of *u*.

The degree *d*(*v*) of a vertex *v* is the number of its incident edges. Let (*e*_1_,…,*e_k_*) be a sequence of edges in a graph. This sequence is called a walk if there are vertices *v*_0_,…,*v_k_* such that *e* = (*v_i_*_−1_,*v_i_*) for *i* = 1,…,*k*, that is the end vertex of an edge *e_i_* is the start vertex of an edge *e_i+_*_1_. If all edges are pairwise distinct and all vertices are pairwise distinct the walk is called a path. The length of a walk or path is given by its number of edges. A shortest path between two vertices *u*, *v* is a path with minimal length. The distance *dist*(*u*,*v*) between two vertices *u*, *v* is the length of a shortest path between them. If no path exists between two vertices *u*, *v*, then the distance *dist*(*u*,*v*) is undefined. Two vertices *u*, *v* of a graph are called strongly connected if there exists a walk from vertex *u* to vertex *v*. If any pair of different vertices of the graph is strongly connected, the graph is called strongly connected.

A subgraph of the graph *G* = (*V*, *E*) is a graph *G_s_* = (*V_s_*, *E_s_*) where *V_s_* ⊆ *V* and *E_s_* ⊆ *E*∩(*V_s_* × *V_s_*). Two graphs *G*_1_ = (*V*_1_, *E*_1_) and *G*_2_ = (*V*_2_, *E*_2_) are isomorphic if there is a one-to-one correspondence between their vertices, and there is an edge directed from one vertex to another vertex of one graph if and only if there is an edge with the same direction between the corresponding vertices in the other graph.

**Figure 1:**
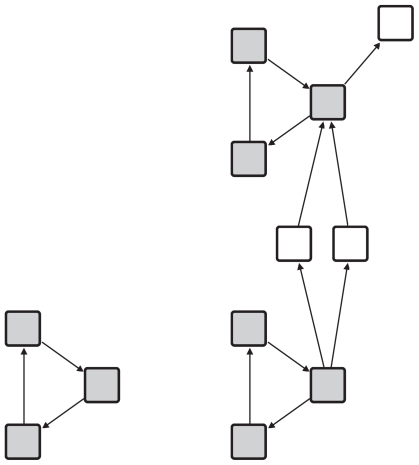
A motif and two matches of the motif in a graph.

Small recurring subgraphs within a given graph are called motifs (Milo et al. (2002)). A motif *M* is a directed graph. A match *G_M_* of a motif *M* in a graph *G* is a subgraph of *G* which is isomorphic to the motif *M*. The motif match set *MS_G_* of a motif *M* is the set of all matches of *M* in the graph *G*. Figure 1 shows a motif and two matches of the motif in a graph.

### Centralities in networks

Formally a centrality is a function *C* which assigns every vertex *v* of a graph a numeric value *C*(*v*). As we are interested in the ranking of the vertices of the given graph *G* we choose the convention that a vertex *u* is more important than another vertex *v* if and only if *C*(*u*) > *C*(*v*).

In the following sections we explain different centrality measures and show an example graph and the corresponding centrality values. We restrict our analysis to centrality measures which have been used to analyze biological networks or are used in our study in the second part of this paper. A comprehensive overview of different centrality measures was published in (Koschützki et al. (2005)).

### Degree centrality

An obvious order of the vertices of a graph can be established by sorting them according to their degree. The corresponding centrality measure degree-centrality is defined as *C_deg_*(*v*) = *d*(*v*). For directed networks two degree centralities, the in-degree centrality (considering only ingoing edges) and the out-degree centrality (considering only outgoing edges), exist. Degree centrality is a local centrality measure: only the immediate neighbourhood of the vertex of interest is considered. Degree can be computed for all kinds of networks. See the work of Freeman (1979) for a list of references to the usage of degree-centrality in social network analysis. For biological network analysis degree centrality has been applied in numerous situations. For example, it is used by Jeong et al. (2001) to correlate the degree of a protein in the network with the lethality of its removal. Another study by Hahn and Kern (2005) compared three centralities (degree, closeness and betweenness) for the identification of essential proteins in three different organisms: *Saccharomyces cerevisiae*, *Caenorhabditis elegans*, and *Drosophila melanogaster*. In all three networks and for all three centralities it was shown that the mean centrality value for essential proteins is significantly higher than the centrality value of nonessential proteins.

### Closeness centrality

Closeness-centrality uses information about the length of the shortest paths within a network; it uses the sum of the minimal distances of a vertex to all other vertices. The closeness-centrality is defined as the reciprocal of this sum: *C_clo_*(*u*) = 1/(∑*_v_*_∈_*_V_dist*(*u*, *v*)). As the distance between vertices is only defined for pairwise strongly connected vertices this centrality can only be applied to strongly connected networks. Closeness-based centrality has been used in different studies. Wuchty and Stadler (2003) apply this centrality to different biological networks and show the correspondence with the service facility location problem. According to a slight modification of the closeness centrality 8 of the top 10 metabolites of the metabolic network of *E. coli* are part of the glycolysis and citrate acid cycle pathways (Ma and Zeng (2003)).

### Radiality and integration

Similar to the closeness measure are the centralities radiality and integration introduced by Valente and Foreman (1998). The computation of both centralities is based on the reverse distance matrix which is defined on the basis of the distance matrix *D* = (*dist*(*i*, *j*)). The reverse distance matrix *RD* is defined as *RD_ij_* = *diameter*(*G*) + 1 − *D_ij_*, where *diameter*(*G*) is the diameter, the highest distance value, of the graph. On the basis of this matrix *RD* radiality is defined as *C_rad_*(*i*) = (∑*_i_*_≠_*_j_* *RD_ij_*)/(*n*−1) and integration is defined as *C_int_*(*j*) = (∑*_i_*_≠_*_j_* *RD_ij_*)/(*n*−1).

A vertex with a high radiality value can easily reach other vertices. A vertex with a high integration value is easily reachable from other vertices. Similarly to closeness both radiality and integration are shortest path based measures. In contrast to closeness which can be only computed for strongly connected networks, radiality and integration can also be computed for weakly connected or even unconnected networks.

### Shortest path betweenness centrality

Shortest path betweenness centrality quantifies the ability of a vertex to monitor communication between other vertices. Every vertex that is part of a shortest path between two other vertices can monitor communication or flow between them. Counting how many such communications a vertex may monitor leads to an intuitive definition of a centrality: a vertex is central if it can monitor many communications between other vertices. In the following let *σ_st_* denote the number of shortest paths between two vertices *s* and *t*, and let *σ_st_*(*v*) denote the number of shortest paths between *s* and *t* that use *v* as an interior vertex. The rate of communication between *s* and *t* that can be monitored by an interior vertex *v* is denoted by δ*_st_*(*v*) = *σ_st_*(*v*)/*σ_st_*. If no shortest path between *s* and *t* exists we set δ*_st_*(*v*) = 0. The shortest path betweenness centrality (Freeman (1977)) is defined as *C_spb_*(*v*) = ∑*_s_*_≠_*_v_*_∈_*_V_*∑*_t_*_≠_*_v_*_∈_*_V_δ_st_* (*v*).

There are several studies investigating shortest path betweenness in biological networks. For an *S. cerevisiae* protein interaction network it was reported that proteins with a high betweenness centrality value cover a broad range of degree centrality values. In particular, proteins with a high betweenness and low degree value (HBLC, high betweenness low connectivity proteins) are prominent as they are supposed to support modularization of the network (Joy et al. (2005)). Shortest-path betweenness centrality was applied to mammalian transcriptional regulatory networks and it was noted that betweenness appears to be an interesting topological characteristic in regard to the biological significance of distinct elements (Potapov et al. (2005)).

### Katz status index and PageRank

For the analysis of gene regulatory networks discussed in the second part two further centralities can be applied: the status index defined by Katz (1953) and the PageRank centrality (Page et al. (1998)) which is the algorithmic method behind the search engine Google. Both centralities are best described as computations performed on the adjacency matrix accompanied to the graph of interest. As we focus on the result of different centralities and their comparison we skip a lengthy formal definition here and refer to the literature for details (Katz (1953); Page et al. (1998); Koschützki et al. (2005); Koschützki (2008)).

### Motif-based centralities

**Figure 2:**
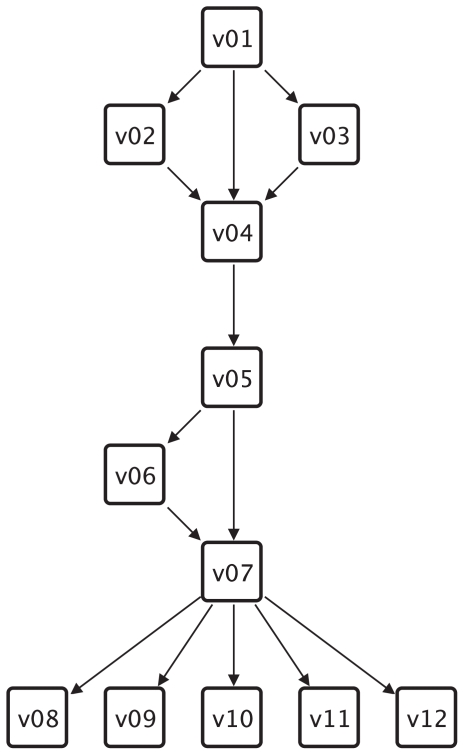
An example graph used to explain different centrality measures.

**Figure 3:**
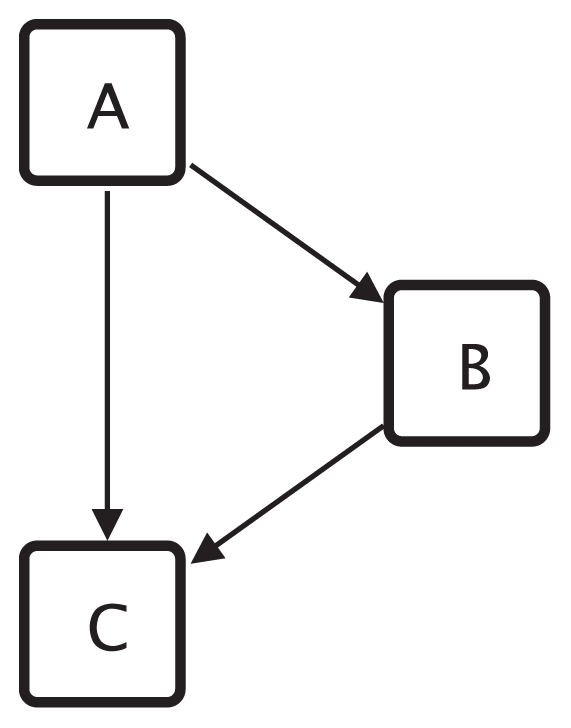
The FFL motif with roles.

Given a graph *G*, a motif *M* and the corresponding motif match set *MS_G_* a centrality can be defined. The motif-based centrality *C_mb_* assigns to every vertex *v* the number of matches the vertex *v* occurs in (Koschützki et al. (2007)). For example the vertex *v*01 in the graph shown in Figure 2 occurs in two matches of the FFL motif shown in Figure 3. Therefore *C_mb_*(*v*01) = 2. Two extensions of this centrality exist: motif-based centrality with roles and motif-based centrality with classes.

Vertices of motifs may represent different functions. For example, in the gene regulatory network context three different functions of the vertices of the feed forward loop (FFL) motif as shown in Figure 3 can be identified: (1) the vertex at the top is the master regulator, this vertex regulates the other two vertices; (2) the vertex on the right side is the intermediate regulator, it is regulated by the master regulator and itself regulates together with the master regulator the vertex at the bottom; and (3) the vertex at the bottom of the drawing is regulated by both other vertices and is therefore called the regulated vertex. Such different functions of vertices within motifs are called roles and three roles can be assigned to the vertices of the FFL motif. The motif-based centrality with roles *C_mbr_* restricts the number of counted matches to those matches where the vertex occurs in the match with the role under consideration; see Koschützki et al. (2007) for details.

**Figure 4:**
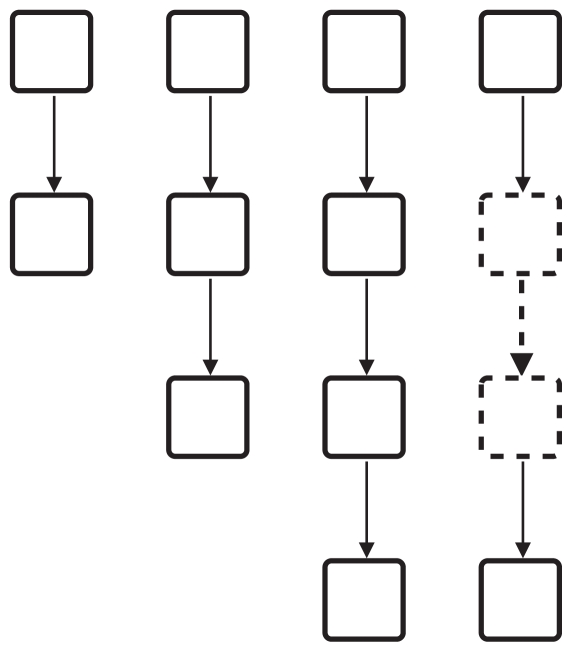
Several motifs of the chain motif class.

Using the previously introduced concepts we can extend the motif-based centrality method further. By assigning the same role to similar vertices of a group of similar motifs we can establish a centrality based on a class (or group) of motifs. Consider, for example, a group of chains (see Fig. 4), where all vertices at the start of such chains have a similar characteristic (no incoming edges) and all vertices at the end have another similar characteristic (no outgoing edges). For gene regulatory networks several motif classes are known. For example, the regulatory chain motif class, as in the example above, consists of a set of chains of three or more regulators in which one regulator regulates another regulator, which in turn regulates a third one and so forth (Lee et al. (2002)). In the motif class single input motif (SIM) a set of vertices is exclusively regulated by a single vertex (Shen-Orr et al. (2002)). The motif-based centrality with classes *C_mbc_* therefore is the sum of motif-based centralities with roles *C_mbr_* for the same role in similar or related motifs.

Several motifs have been studied in all kinds of biological networks. The best studied motif is the FFL motif which functional properties have been analyzed in detail theoretically and experimentally especially in gene regulatory networks (Mangan and Alon (2003); Mangan et al. (2003); Shen-Orr et al. (2002); Wall et al. (2005)). However, in these approaches only the occurrence of motifs is considered but motifs are not used to rank the genes.

Different motifs occurring in a human cellular signalling network were analysed by Awan et al. (2007). They discovered that genes which are related to cancer are enriched in the target vertices of several motifs and that cell mobility genes are enriched in the source vertices of motifs. For a gene regulatory network of *E. coli* Wang and Purisima (2005) discovered, that transcript with short half-lives are enriched in motifs, especially in SIMs, FFLs and bi-fans.

### Example graph and centralities

Figure 2 shows a small example graph and the corresponding Table 1 shows the centrality values for the centralities that are applicable to this graph.

## Analysing Gene Regulatory Networks with Centralities

The applicability of specific centrality measures for the investigation of biological networks depends on the type of the particular network, and depending on the type of the network different centrality measures are used. Here we focus our analysis on gene regulatory networks.

As an example, we analyze centralities within the gene regulatory network (GRN) of *Escherichia coli*. The network is based on the data of transcriptional regulatory interactions of genes from RegulonDB, Version 5.5 (Salgado et al. (2006)). Genes are represented by vertices and transcriptional regulatory interactions between genes are modelled as edges, a common approach to model GRNs. The interactions between genes represent transcriptional control of transcription factors on the transcription of regulated genes. There are a few cases where transcription factors are formed by subunits of different gene products. They are here replaced by a common identifier which corresponds to the transcription factor, e.g. *ihfA* or *ihfB* result in *ihfAB*. The regulatory interactions of such different subunits are assigned to this new identifier, and parallel edges which occurred due to the previous operation are replaced by a single edge. The resulting network consists of 1250 vertices and 2515 edges. In gene regulatory networks genes at a high level within the hierarchy of regulatory control are of particular interest due to their far reaching influence on other genes within the network. These genes are commonly called global regulators. Some criteria for the characterization of global regulators have been proposed, such as the number of regulated genes, the number and type of co regulators, the number of other regulators they control, the size of their evolutionary family, and the variety of conditions where they exert their control (Martínez-Antonio and Collado-Vides (2003)).

### Comparison of different centralities for GRN

In this section, we compare different centrality measures that can be applied to GRNs. As GRNs are directed graphs that are not necessarily strong connected only the centralities degree, shortest-path betweenness, integration, radiality, Katz status index, PageRank and the different motif-based centralities can be applied. The centralities PageRank and Katz status index are sensible to the directionality of the edges and therefore we consider two variants of the graph, the original graph and the graph with all edge directions reversed.

The top 25 genes (top 2% of all genes) according to the eight best centrality measures (i.e. the centrality measures which identify the highest number of global regulators within the top 2% of all genes) are shown in Table 2. In total 18 global regulators have been identified by Martínez-Antonio and Collado-Vides (2003). All different centrality measures shown in Table 2 are able to identify more than 50% of the global regulators within the top 2% of the ranked genes. For example, shortest path betweenness finds 11 global regulators and motif-based centrality with the chain motif class is able to identify 15 global regulators.

It should be also noted that for nearly all centrality measures the top 5 positions are occupied by global regulators. However, all centralities result in different rankings even for global regulators which are often ranked very high. For example, the gene *ihfAB* is ranked either very high at the second position (e.g. radiality, PageRank) or not even under the top 25 genes (shortest path betweenness). Radiality ranks similar to the motif-based centrality with the chain motif class (short chain centrality) but even in this short list differences are visible. For example, the global regulator *fur* ranked on position 8 (radiality) is ranked on position 18 by the chain centrality.

Correlation coefficients are a valid measure to show that centralities do not coincide. Table 3 shows the pairwise Kendall’s correlation coefficients for the centralities used in Table 2. From these centralities only a few correlate with a coefficient above 0.9 to other centralities. These are out-degree, PageRank, Katz status index, radiality and the motif-based centrality with chain classes (chain). The centralities based on the FFL motif and shortest-path betweenness do correlate only with correlation coefficients less than 0.9 to other centralities.

For the five centralities with a correlation coefficient above 0.9 these high coefficients can easily be explained: 1101 out of 1250 (88.08%) vertices have an out-degree of zero. All these vertices are assigned the same centrality value of nearly zero for the Katz status index and the PageRank centrality, and the value zero for the radiality and the motif-based centrality with chain classes. Therefore, the comparison of correlations between all centrality values is not feasible for the complete vector of centralities: all five centralities rank these 1101 vertices into the same group.

Table 4 shows the pairwise correlation coefficients for the centrality values of the vertices which have a non-zero out-degree. These coefficients show a different picture: all five centralities do rank the remaining 149 genes differently, only the centrality radiality and Katz status index archive a considerable high correlation to each other and to the motif-based centrality with chain classes.

In conclusion, the centralities applied to the GRN rank the genes differently and the motif-based centrality with chain classes is able to rank the highest number of interesting genes (global regulators) within the top 2% of all genes. The chain centrality identifies 15 out of 18 global regulators (83%) identified by Martínez-Antonio and Collado-Vides (2003) and outperforms the other centralities used.

## Discussion

To investigate large biological networks different analysis methods have been developed, and centrality analysis is a particularly useful method to analyze the structure of these networks. In this paper we discussed and compared different centrality measures and applied them to a gene regulatory network of *E. coli*. The results show that using centrality analysis methods from other fields of sciences such as social network analysis is a starting point to investigate gene regulatory networks. However, we also show that it is useful to consider biological knowledge in network analysis and that the recently introduced motif-based centrality outperforms other methods.

The comparison of the pairwise correlation coefficients and the analysis of the rankings of the top 25 genes show that the motif-based centralities, in particular with the chain motif class, produce rankings different to the rankings computed by existing centralities, and that these rankings show interesting features of the gene regulatory network under analysis.

## Figures and Tables

**Table 1:** The centrality values that are discussed in this paper computed for the example graph in Figure 2.

	ideg	odeg	par	parR	kat	katR	spb	int	rad	chains	fflA	fflB	fflC	fflSum
**v01**	0.00	3.00	0.04	0.19	0.00	37.64	0.00	0.00	2.18	47.00	2.00	0.00	0.00	2.00
**v02**	1.00	1.00	0.05	0.07	0.95	12.32	0.00	0.36	1.45	15.00	0.00	1.00	0.00	1.00
**v03**	1.00	1.00	0.05	0.07	0.95	12.32	0.00	0.36	1.45	15.00	0.00	1.00	0.00	1.00
**v04**	3.00	1.00	0.12	0.16	4.66	11.97	24.00	1.09	1.82	14.00	0.00	0.00	2.00	2.00
**v05**	1.00	2.00	0.14	0.16	5.37	11.60	28.00	1.18	2.09	13.00	1.00	0.00	0.00	1.00
**v06**	1.00	1.00	0.10	0.08	6.05	5.46	0.00	1.18	1.73	6.00	0.00	1.00	0.00	1.00
**v07**	2.00	5.00	0.18	0.14	12.75	4.75	30.00	1.55	1.82	5.00	0.00	0.00	1.00	1.00
**v08**	1.00	0.00	0.07	0.03	13.07	0.00	0.00	1.36	0.00	0.00	0.00	0.00	0.00	0.00
**v09**	1.00	0.00	0.07	0.03	13.07	0.00	0.00	1.36	0.00	0.00	0.00	0.00	0.00	0.00
**v10**	1.00	0.00	0.07	0.03	13.07	0.00	0.00	1.36	0.00	0.00	0.00	0.00	0.00	0.00
**v11**	1.00	0.00	0.07	0.03	13.07	0.00	0.00	1.36	0.00	0.00	0.00	0.00	0.00	0.00
**v12**	1.00	0.00	0.07	0.03	13.07	0.00	0.00	1.36	0.00	0.00	0.00	0.00	0.00	0.00

**Abbreviations:** chains: motif-based centrality for the chain class; fflA, fflB and fflC: motif-based centrality for the FFL motif with roles (different roles A, B, C; see Figure 3); fflSum: motif-based centrality for the FFL motif without roles; ideg: in-degree; int: integration; kat: Katz status index; katR: Katz status index for the reversed graph; odeg: out-degree; par: PageRank; parR: PageRank for the reversed graph; rad: radiality; spb: shortest-path betweenness.

**Table 2:** Names of the top 25 genes (top 2% of all genes) according to 8 best centrality measures, i.e. centralities which find a high number of global regulators within the top 2% of all genes. Global regulators according to Martínez-Antonio and Collado-Vides (2003) are highlighted in bold face. Note that in few cases were genes with the same centrality value occur they are ranked in alphabetical order. For each centrality the last row of the table shows the number of global regulators identified within the top 2% of all genes.

position	odeg	parR	katR	spb	rad	chains	fflA	fflSum
1	***crp***	***crp***	***crp***	***hns***	***crp***	***crp***	***crp***	***crp***
2	***fnr***	***ihfAB***	***fnr***	*gadX*	***ihfAB***	***ihfAB***	***fnr***	***fnr***
3	***ihfAB***	***fnr***	***arcA***	*flhD*	***fnr***	***arcA***	***ihfAB***	***arcA***
4	***fis***	***arcA***	***ihfAB***	***fur***	***arcA***	***fnr***	***arcA***	***fis***
5	***arcA***	***phoB***	***fis***	*gadE*	***fis***	***fis***	***fis***	***narL***
6	***narL***	*lexA*	***hns***	***fis***	*gadE*	*evgA*	*modE*	***ihfAB***
7	***hns***	***cpxR***	*gadE*	***lrp***	***hns***	*ydeO*	***soxS***	***hns***
8	***fur***	***soxR***	*gadX*	*rcsAB*	***fur***	*gadE*	***hns***	***fur***
9	***lrp***	***fis***	***cspA***	***soxS***	***soxS***	***soxR***	***cpxR***	*gadX*
10	*glnG*	*evgA*	*evgA*	***fnr***	*evgA*	***soxS***	*fhlA*	*hyfR*
11	*narP*	*cysB*	*ydeO*	***cspA***	*ydeO*	*torR*	*gadE*	*marA*
12	***cpxR***	*argR*	*torR*	*caiF*	*oxyR*	*gadW*	***rob***	*flhD*
13	***phoB***	*phoP*	*gadW*	***purR***	*gadX*	*cspE*	*gadX*	*nagC*
14	*fruR*	***fur***	*cspE*	***narL***	***cspA***	***cspA***	*galR*	***soxS***
15	*modE*	*allR*	***soxS***	*marA*	***narL***	*gadX*	***fur***	*modE*
16	*fhlA*	*glnG*	***soxR***	*metJ*	*modE*	***hns***	*gntR*	*tdcA*
17	*lexA*	*sdaR*	***rob***	*malT*	***soxR***	*oxyR*	*oxyR*	*yiaJ*
18	*flhD*	*trpR*	*marA*	***arcA***	*torR*	***fur***	*tdcR*	*gutM*
19	*gadE*	*agaR*	*marR*	*glnG*	*gadW*	*modE*	*gutM*	***ompR***
20	***purR***	*gadE*	*oxyR*	***ompR***	*cspE*	***narL***	*nagC*	*srlR*
21	***soxS***	***soxS***	***fur***	*Nac*	***lrp***	***lrp***	***narL***	*galS*
22	*argR*	***hns***	*modE*	*oxyR*	*glnG*	*glnG*	***ompR***	*idnR*
23	*cysB*	***lrp***	*gutM*	*hupAB*	***phoB***	***ompR***	*srlR*	*caiF*
24	*marA*	*tyrR*	*srlR*	*argP*	*narP*	***phoB***	*argP*	*chbR*
25	*nagC*	*torR*	***narL***	*dnaA*	***ompR***	***cpxR***	*cysB*	***cpxR***
#global regs.	13	12	12	11	14	15	12	11

**Abbreviations:** see Table 1.

**Table 3:** Kendall’s correlation coefficients for the centralities used in the analysis of the ***E. coli*** network.

	odeg	parR	katR	spb	rad	chains	fflA	fflSum
**odeg**	1	0.97	0.93	0.49	0.98	0.98	0.47	0.17
**parR**	0.97	1	0.92	0.48	0.96	0.96	0.46	0.16
**katR**	0.93	0.92	1	0.47	0.95	0.95	0.46	0.14
**spb**	0.49	0.48	0.47	1	0.49	0.49	0.43	0.22
**rad**	0.98	0.96	0.95	0.49	1	1	0.48	0.18
**chains**	0.98	0.96	0.95	0.49	1	1	0.48	0.18
**fflA**	0.47	0.46	0.46	0.43	0.48	0.48	1	0.29
**fflSum**	0.17	0.16	0.14	0.22	0.18	0.18	0.29	1

**Abbreviations:** see Table 1.

**Table 4:** Kendall’s correlation coefficient for the dataset with the zero out-degree vertices removed.

	odeg	rad	katR	parR	chains
**odeg**	1	0.75	0.7	0.52	0.72
**rad**	0.75	1	0.94	0.51	0.96
**katR**	0.7	0.94	1	0.48	0.97
**parR**	0.52	0.51	0.48	1	0.5
**chains**	0.72	0.96	0.97	0.5	1

**Abbreviations:** see Table 1.
